# Genitalia burn: accident or violence? Concerns that transcend injury treatment

**DOI:** 10.1590/0103-0582201432213713

**Published:** 2014-06

**Authors:** Ana Lúcia Ferreira, Juliana Montez Ferreira, Paula Marques C. da Silva, Dilene Francisco Constancio

**Affiliations:** 1 UFRJ, Rio de Janeiro, RJ, Brasil

**Keywords:** sodium hydroxide, accident prevention, chemical accidents, accidents, home, sexual violence, negligence, child

## Abstract

**OBJECTIVE:**

To describe a case of genital burn which raised the suspicion of maltreatment (sexual abuse and neglect by lack of supervision).

**CASE DESCRIPTION:**

An infant was taken to the Emergency Room of a pediatric hospital with an extensive burn in the vulva and perineum. The mother claimed the burn had been caused by a sodium-hydroxide-based product. However, the injury severity led to the suspicion of sexual abuse, which was then ruled out by a multidisciplinary team, based on the consistent report by the mother. Besides, the lesion type matched those caused by the chemical agent involved in the accident and the family context was evaluated and considered adequate. The patient had a favorable outcome and was discharged after four days of hospitalization. Outpatient follow-up during six months after the accident enabled the team to rule out neglect by lack of supervision.

**COMMENTS:**

Accidents and violence are frequent causes of physical injuries in children, and the differential diagnosis between them can be a challenge for healthcare workers, especially in rare clinical conditions involving patients who cannot speak for themselves. The involvement of a multidisciplinary trained team helps to have an adequate approach, ensuring child protection and developing a bond with the family; the latter is essential for a continued patient follow-up.

## Abstract (es)

**OBJETIVO:**

Describir un caso de quemadura de los genitales que llevó a la sospecha de malos tratos (abuso sexual y negligencia de supervisión).

**DESCRIPCIÓN DEL CASO:**

Lactante fue llevada a la Emergencia de un hospital pediátrico por lesión extensa en región de la vulva y entre la vagina y el ano. La madre alegaba quemadura por producto a base de soda cáustica, pero la gravedad de la lesión llevó el equipo a sospechar de abuso sexual. La sospecha fue alejada por equipo multidisciplinario con base en el relato consistente de la madre, en el tipo de lesión compatible con el agente químico implicado en el accidente y en la evaluación del contexto de vida familiar. El cuadro clínico evolucionó de manera satisfactoria, siendo dada alta después de cuatro días de internación. El seguimiento ambulatorial en los seis meses posteriores permitió alejar también una posible situación de negligencia de supervisión, con base en la historia previa del niño, en su estado de salud y en la observación de los vínculos familiares existentes y de los cuidados dispensados por la familia a lo largo del tiempo.

**COMENTARIOS:**

Accidentes y violencia son causas frecuentes de lesiones físicas en niños, siendo el diagnóstico diferencial de ambos un desafío para los profesionales, especialmente frente a cuadros clínicos raros en pacientes que todavía no verbalizan. La actuación de equipo multidisciplinario con profesionales instrumentalizados para ese tipo de atención ayuda en la conducción adecuada, garantizando la protección del niño y la creación de vínculo con la familia, lo que es esencial para un posterior seguimiento que haga viable la observación continuada del caso.

## Introduction

External causes of morbidity and mortality (i.e., accidents and violence) in children and adolescents are a public health problem in Brazil. In 2011, in the state of Rio de Janeiro, 511 children between one and four years old died, mostly due to external causes (126 cases)^(^ 1 ^)^. These events are important because of both the frequency with which they affect this age group and their short- and long-term consequences. It should be noted that only a small portion of these occurrences result in death.

Burns, whether accidental or intentional, are very common in children and usually occur at home. Several previous studies have analyzed childhood chemical burns resulting from the accidental ingestion of alkalis^(^ 2 ^,^ 3 ^)^. Children under six are at the greatest risk of poisoning with caustic products in the home environment in developing countries^(^ 4 ^)^. Conversely, there are few reports of children with skin injuries resulting from contact with such products. In the United States, skin contact is responsible for one third of injuries caused by cleaning products in children; also in that country, intake is the most frequent mechanism of injury^(^ 5 ^)^.

In Brazil, statistical data have shown that chemical agents are the cause of only 1 to 4% of burns, with caustic soda being the major causative agent^(^ 6 ^)^. In a study conducted in Rio de Janeiro, skin exposure to cleaning products in the home environment accounted for only 2.4% of cases^(^ 7 ^)^. Despite this low frequency, health teams should be prepared to deal with this problem, because of the severe clinical consequences involved.

Another challenge in the pediatric care of injuries by external causes is the difficulty involved indistinguishing between accidental and intentional injuries. This problem becomes even greater when dealing with infants, as it is not possible to rely on the patient's ability to talk.

According to the Brazilian Society of Pediatrics^(^ 8 ^)^, and similarly to other types of accidents, most burns do not occur by chance, but rather result from neglect (failure to protect and/or teach children) or constitute a cruel form of abuse (deliberate injury). The latter situation accounts for 10% of the various forms of abuse and for 1 to 16% of the total number of burned children and adolescents who seek medical assistance (p. 135).

The unusual case of genital burn by caustic soda here reported aims to discuss issues that go beyond clinical care and that can help healthcare teams to distinguish an accidental event from suspected maltreatment (sexual abuse and supervisory neglect).

The patient's medical record was reviewed to collect data on her admission to the emergency department and subsequent outpatient appointments at the Clinic of General Pediatrics in the Martagão Gesteira Institute of Child Care and Pediatrics (Instituto de Puericultura e PediatriaMartagão Gesteira, IPPMG), at Universidade Federal do Rio de Janeiro (UFRJ).

## Case description

**Figure 1:**
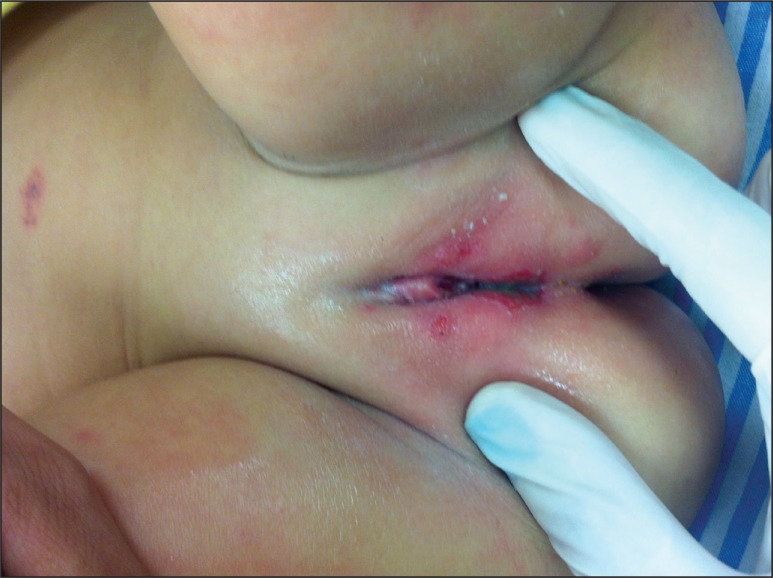
Deep burn injury affecting the vulvar region and a lesion on the abdomen.

**Figure 2:**
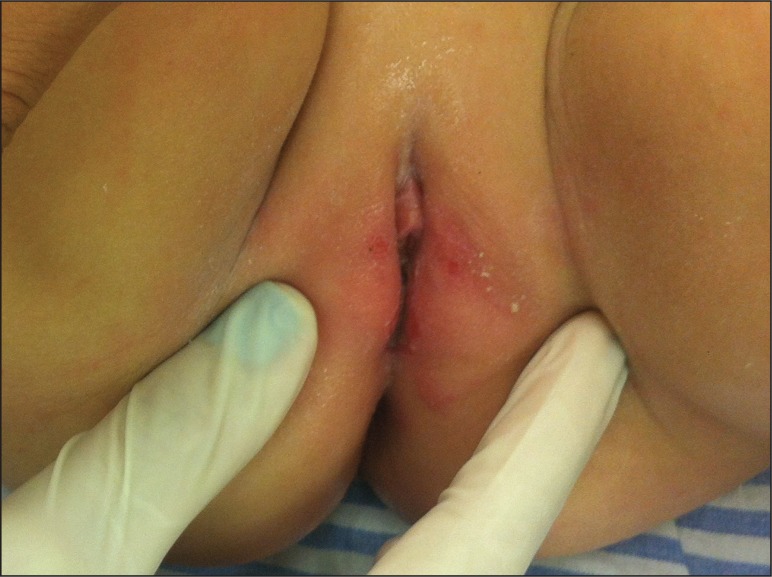
Burn on the outer part of the vulvar region

A 21-month-old female was brought by her mother to the emergency department of the hospital. The mother reported that, upon waking up, approximately 90 minutes prior to her arrival at the hospital, she found the infant next to her on the bed carrying a caustic soda container. The infant would have had access to the container while the mother was asleep. The mother then realized that the substance was present in her daughter's vulvar region, and reported having thoroughly washed it with water and then sought medical care. Upon clinical examination, the child was in good general condition and active. She presented a burn injury affecting the vulvar region and the perineum, with a necrotic area of approximately 2cm in diameter, a bilateral second degree burn of approximately 0.5cm in diameter, and hyperemia in the surrounding area (Figures 1 and 2). The region was cleaned with 0.9% saline and moisturized with a dermatological oil, according to guidelines from the Rio de Janeiro Intoxication Center. Pediatric surgery and gynecology services were consulted, and the case was referred to the social service due to suspicion of sexual abuse. The social worker who evaluated the patient and her family is part of a multidisciplinary team - also comprising pediatricians, psychologists, and nurses - that provides assistance to families experiencing violence situations. The case was analyzed by the team soon after the patient was admitted to the emergency department. The child remained hospitalized.

On the following day, the mother told the gynecologist that she generally used talcum powder when changing her daughter's diapers, and believed that the infant had mistakenly used caustic soda on her vulva, thinking it was talcum powder. Examination showed slight improvement of the genital injury, absence of active bleeding, and an intact hymen. During physical examination, the child imitated gestures made by the team, corroborating the mother's account that the girl used to imitate gestures. The gynecology service concluded that the injuries were consistent with maternal report and with burn caused by caustic soda.

At an appointment with the social service, on the same day, the mother reported that the substance causing the injury was a cleaning product used to unclog sinks. The product was kept in the kitchen, on the floor, behind the stove, so that the infant could not reach it. The family was from the state of Ceará and had been living in Rio de Janeiro for a few months. The mother was 19 years old and worked as a waitress at night. The father was 20 years old and worked as a kitchen assistant in the morning. Both parents took turns in taking care of the infant during the day, with occasional help from other family members. The infant did not attend daycare.

Maternal report and physical examination failed to confirm the multidisciplinary team's suspicion of sexual abuse. As a result, the case was considered to be an accident, and was not notifiedto the Guardianship Council. 

The patient was discharged after four days of hospitalization. During outpatient follow-up, the team considered it appropriate to learn more about the circumstances of the accident so as to assess the possibility of supervisory neglect. The mother confirmed the same facts reported previously, adding that, before washing the region, she had tried to remove the product from the infant's vulva by rubbing it with her hand to "clean" it. She felt guilty because she found out, at the hospital, that this action may have contributed to worsen the burn. Subsequent contact with the family after discharge did not suggest care neglect.

## Discussion

Sodium hydroxide (caustic soda) is a solid, non-combustible, hygroscopic substance, highly soluble in water; the aqueous solution is highly alkaline^(^ 9 ^)^. Caustic soda is found in some household cleaning products, e.g., those used to unclog sinks and drains; therefore, it is commonly within reach of children, as in the case here reported.

Alkali burns penetrate deeper in the skin when compared to thermal or acid burns. This type of tissue injury comprises three factors: intense cellular dehydration; saponification of fat, leading to the loss of thermal insulation; and enzyme protein inactivation and binding with alkali, resulting in a chemical reaction that releases heat and further aggravates the initial injury^(^ 6 ^,^ 10 ^)^. Severity of tissue damage is directly related to substance type, amount, and concentration, as well as to the duration of contact with skin. Alkali burns can have acute or chronic complications, or even permanent sequelae^(^ 10 ^)^.

Chemical burns in children are primarily accidental. In the literature, only two case reports of exposure to caustic soda as a result of children maltreatment were found^(^ 11 ^,^ 12 ^)^.

The rare frequency of injuries caused by caustic soda, on the one hand, and the extent and depth of the injury reported in this study, on the other, were the main reasons behind the emergency team's suspicion of sexual abuse. It was considered unlikely that the mere contact with a cleaning product, as reported by the mother, would have been able to cause the injury found. In general, controversy between the type of injury observed and the causative mechanism reported by parents is one of the indicators justifying suspicion of violence, and requires a thorough evaluation to clarify the circumstances leading to injury^(^ 13 ^)^.

Whenever there is the possibility that the injury may have been intentional, professionals have to deal with other issues - in the present report, suspicion of sexual abuse and the possibility of negligent supervision of the child. In both situations, the healthcare team has to go beyond merely clinical aspects to clarify and understand the social, cultural, and emotional background of the families seeking assistance. This approach requires, on the part of healthcare professionals, personal availability for an attentive listening and some degree of experience in handling difficult cases calmly, not scaring or accusing the family.

Professionals dealing with these situations also need to clarify the circumstances that have caused the injury, find out who takes care of the child, and whether this care is appropriate for his or her age and development. It is also useful to learn about the environment where the family lives, whether other accidents have already occurred, whether there has been a delay in seeking care, and whether the child behaves and interacts appropriately with the caregiver^(^ 14 ^)^. These data will make it possible to assess the level of risk to which the child may be exposed and will help decide on how to manage the case, e.g., hospitalization as a protective measure, notification of the Guardianship Council,or outpatient follow-up. It is worth mentioning that notification should be made even in cases of suspicion whenever it is not possible to involve other professionals.

Because the emergency department is an important route of admission to the health system, professionals working in this setting should be able to develop positive rapport with guardians, so as to help increase adherence to the follow-up treatment proposed after discharge. Outpatient follow-up is a way of ensuring child protection, as it allows long-term monitoring of the case. For instance, it allows to analyze the bond between child and parents, the family characteristics, and new health problems that may emerge. Finally, follow-up is a way of providing guidance to parents regarding the prevention of accidents and maltreatment.

Neglect is a very common type of violence against children worldwide. According to the Brazilian Ministry of Health, neglect is a disregard for the welfare, safety, affection, or education of children or adolescents, or the parents' refusal to follow guidance regarding the immunization schedule, drug treatments, and educational and preventative guidelines^(^ 13 ^)^. The chronicity and recurrence of these acts are important aspects to be taken into consideration when determining the presence of neglect.

As for the case reported here, social family history, family ties, and previous care of the child indicated that she was receiving adequate care and that the accident was the result of an isolated oversight. During outpatient follow-up, the mother argued that she usually closed the bedroom door whenshe and her daughter slept in the afternoon, but she had forgotten to do so that day. The outpatient team focused on advising the mother to require family help in the care of the child and increase accident prevention measures. Over six months after the accident, adherence to follow-up was good, and no complications were observed. 

In order to promote the safety of infants aged between one and two years, it is important to advise parents to keep sharp objects, or objects that can be swallowed, out of children's reach; to use protection devices to prevent falls on stairs or from windows; to use edge and corner protectors on furniture; to use a gate or other obstacle to prevent access to the kitchen door and keep the bathroom door closed; to use special child seats in the car, always in the back seat; and to store cleaning products and medicines in locked, high cabinets^(^ 15 ^)^. 

The present case deserves the attention of healthcare professionals because it underscores the importance of accident prevention, a topic that should be consistently emphasized in the follow-up of children and adolescents and adapted according to the stages of the child's development and the family's way of living. Health teams should be prepared to consider violence as a differential diagnosis whenever necessary and should know how to clarify suspicions in a welcoming manner, not to undermine either the care process itself or the relationship with the family.
