# Supplementary material for: Genitalia burn: accident or violence? Concerns that transcend injury treatment
Source: Rev Paul Pediatr. 2014 Jun;32(2):286–90. doi: 10.1590/0103-0582201432213713 (PMC4183022; doi:10.1590/0103-0582201432213713)
Supplement: Translation

# Translation: Queimadura de genitália: acidente ou violência? Preocupações que transcendem o tratamento das lesões

**Authors:** Ana Lúcia Ferreira, Juliana Montez Ferreira, Paula Marques C. da Silva, Dilene Francisco Constancio

**Affiliations:** 1 UFRJ, Rio de Janeiro, RJ, Brasil

**Keywords:** hidróxido de sódio, prevenção de acidentes, acidentes químicos, acidentes domésticos, violência sexual, negligência, criança

## Abstract

**OBJETIVO:**

Descrever um caso de queimadura de genitália que levou à suspeita de maus-tratos (abuso sexual e negligência de supervisão).

**DESCRIÇÃO DO CASO:**

Lactente foi levada à Emergência de um hospital pediátrico por lesão extensa em região vulvar e entre a vagina e o ânus. A mãe alegava queimadura por produto à base de soda cáustica, mas a gravidade da lesão levou a equipe a suspeitar de abuso sexual. A suspeita foi afastada por equipe multidisciplinar com base no relato consistente da mãe, no tipo de lesão compatível com o agente químico envolvido no acidente e na avaliação do contexto de vida familiar. O quadro clínico evoluiu satisfatoriamente, sendo dada alta após quatro dias de internação. O acompanhamento ambulatorial nos seis meses posteriores permitiu afastar também uma possível situação de negligência de supervisão.

**COMENTÁRIOS:**

Acidentes e violência são causas frequentes de lesões físicas em crianças, sendo o diagnóstico diferencial de ambos um desafio para os profissionais, especialmente diante de quadros clínicos raros em pacientes que ainda não verbalizam. A atuação de equipe multidisciplinar com profissionais instrumentalizados para esses atendimentos ajuda na condução adequada, garantindo a proteção da criança e a criação de vínculo com a família, o que é essencial para o acompanhamento continuado do caso.

## Introdução

As causas externas de morbimortalidade (acidentes e violência) em crianças e adolescentes representam um problema de Saúde Pública no nosso país. Em 2011, no Estado do Rio de Janeiro, ocorreram 511 óbitos de crianças de um a quatro anos de idade, sendo grande parte (126) por causas externas^(^ ^1^ ^)^. Esses eventos são importantes, tanto pela frequência com que afetam a população nessa faixa etária, quanto pelas consequências em curto e longo prazos, sendo os óbitos a expressão de apenas pequena parcela das ocorrências. 

As queimaduras são muito frequentes em crianças. Nessa faixa etária, elas ocorrem geralmente no lar e podem ser acidentais ou intencionais. Na literatura, frequentemente encontram-se artigos sobre queimaduras químicas na infância decorrentes da ingestão acidental de álcalis^(^ ^2^ ^,^ ^3^ ^)^, sendo as crianças menores de seis anos as que apresentam maior risco para o envenenamento cáustico no ambiente doméstico em países em desenvolvimento^(^ ^4^ ^)^. Por outro lado, não são comuns os relatos de lesões de pele decorrentes de contato de crianças com tais produtos. Nos Estados Unidos, o contato é o mecanismo responsável por um terço das lesões causadas em crianças por produtos de limpeza, sendo a ingestão também o mecanismo mais frequente^(^ ^5^ ^)^.

No Brasil, estatísticas mostram que apenas 1 a 4% das queimaduras são provocadas por agentes químicos, tendo a soda cáustica como um dos principais causadores^(^ ^6^ ^)^. Em estudo realizado no Rio de Janeiro, a via dérmica de exposição a produtos saneantes em ambiente doméstico correspondeu a apenas 2,4% dos casos^(^ ^7^ ^)^. Apesar da pouca frequência, a gravidade das consequências clínicas aponta para a necessidade de as equipes de saúde estarem preparadas para lidar com esse problema.

Outro desafio é que, nos atendimentos pediátricos por causas externas, é comum a dificuldade de se distinguirem os mecanismos acidentais dos intencionais. Em lactentes, esse problema torna-se ainda maior, uma vez que não se pode contar com a verbalização do paciente. 

Segundo a Sociedade Brasileira de Pediatria^(^ ^8^ ^)^, "como ocorre nos demais acidentes, na maioria das vezes, as queimaduras não acontecem fortuitamente, mas resultam da negligência (falha na proteção e/ou educação da criança) ou constituem uma cruel forma de abuso (lesão deliberada). Esta última situação representa 10% dentre as várias formas de abuso e de 1 a 16% do número global de crianças e adolescentes queimados que procuram assistência médica" (p. 135).

Este relato de caso, incomum, de queimadura de genitália por soda cáustica, visa discutir aspectos que transcendem a assistência clínica e que podem auxiliar a equipe de saúde a distinguir um evento acidental da suspeita de maus-tratos (abuso sexual e negligência de supervisão). 

Revisou-se o prontuário da paciente, coletando-se dados da internação no setor de Emergência e das consultas ambulatoriais subsequentes no Ambulatório de Pediatria Geral do Instituto de Puericultura e Pediatria Martagão Gesteira (IPPMG), da Universidade Federal do Rio de Janeiro (UFRJ).

## Descrição do caso

Paciente de 21 meses, sexo feminino, foi trazida pela mãe ao setor de Emergência do hospital. Esta relatava que, ao acordar, há aproximadamente 90 minutos, percebeu que a criança estava ao seu lado, sobre a cama, com o pote de uma substância à base de soda cáustica. A lactente teria tido acesso ao frasco do produto enquanto a mãe dormia. Ao perceber que a menina apresentava parte da substância na região da vulva, a mãe relatou ter lavado a lesão com água em abundância e ter buscado atendimento médico. Ao exame, a criança encontrava-se em bom estado geral e ativa. Observou-se queimadura em região vulvar e entre a vagina e o ânus, com área de necrose medindo aproximadamente 2cm de diâmetro, queimadura de segundo grau de aproximadamente 0,5cm de diâmetro, bilateral e hiperemia ao redor (Figuras 1 e 2). Foi feita a limpeza local com soro fisiológico 0,9% e aplicado óleo de uso dermatológico, seguindo-se orientação do Centro de Intoxicações do Rio de Janeiro. Solicitou-se parecer aos serviços de Cirurgia Pediátrica e Ginecologia Infantil, comunicando-se o caso ao Serviço Social pela suspeita de abuso sexual. A assistente social que avaliou a paciente e sua família faz parte de uma equipe multidisciplinar que acompanha famílias em situação de violência no IPPMG (na qual há pediatras, psicólogas e enfermeira), tendo levado o caso para a avaliação da referida equipe logo após a entrada na emergência. A criança permaneceu internada.

**Figura 1:** Queimadura na região vulvar, sendo possível identifi car a profundidade das lesões e uma lesão em abdome

**Figura 2:** Queimadura na parte externa da região vulvar

No dia seguinte, a mãe relatou à ginecologista que tinha o hábito de usar talco na criança a cada troca de fraldas e acreditava que a filha teria confundido a substância com talco, passando-a na vulva. Ao exame, observou-se discreta melhora da lesão de genitália, ausência de sangramento ativo e hímen íntegro. Durante o exame físico, a criança reproduziu gestos da equipe, corroborando o relato da mãe de que ela repetia seus gestos. A conclusão do serviço de Ginecologia foi de que as lesões encontradas eram compatíveis com o relato materno e com queimadura pela substância química envolvida.

No atendimento realizado pelo Serviço Social, também no dia seguinte, a responsável relatou que a substância em questão era um produto de limpeza utilizado pela família para desentupir pias, sendo guardada no chão da cozinha da casa, atrás do fogão, para que a criança não tivesse acesso ao produto. A família era procedente do Ceará e residia há poucos meses na cidade do Rio de Janeiro. A mãe, 19 anos, garçonete, trabalhava no turno da noite. O pai, 20 anos, trabalhava como auxiliar de cozinha no turno da manhã. Ambos se revezavam nos cuidados diários com a criança, com o suporte eventual de familiares. A criança não frequentava creche.

A impressão da equipe multidisciplinar, com base no relato materno complementado pelo exame físico, não reforçou as suspeitas de possível ocorrência de abuso sexual. Assim, considerou-se o fato como acidente, optando-se pela não notificação ao Conselho Tutelar naquele momento. 

Após quatro dias de internação hospitalar, a paciente recebeu alta. No acompanhamento ambulatorial, percebeu-se a necessidade de esclarecer melhor as circunstâncias do acidente para avaliar se teria havido negligência de supervisão. A mãe confirmou os mesmos fatos, acrescentando que, antes de lavar, tentou retirar o produto da vulva da criança esfregando sua mão para "limpar". Sentia-se culpada porque soube, já no hospital, que essa ação pode ter contribuído para tornar a queimadura mais profunda. Os contatos com a família após a alta não sugeriram negligência de cuidados. 

## Discussão

O hidróxido de sódio (soda cáustica) é substância sólida incombustível, higroscópica e bastante hidrossolúvel, cuja solução aquosa é fortemente alcalina^(^ ^9^ ^)^. Compõe produtos de limpeza domésticos, como aqueles usados para desentupir pias e ralos, o que faz com que crianças fiquem mais expostas ao contato, como no caso relatado. 

As queimaduras por álcalis penetram mais profundamente na pele, se comparadas às queimaduras térmicas ou por ácidos. Essa lesão tecidual compreende três fatores: desidratação celular intensa; saponificação da gordura, levando à perda do isolamento térmico do corpo; e a inativação das proteínas enzimáticas que, paralelamente, formam ligações com o álcali, originando uma reação com produção de calor, agravando ainda mais a lesão inicial^(^ ^6^ ^,^ ^10^ ^)^. A gravidade dessas lesões teciduais está diretamente relacionada ao tipo, à quantidade e à concentração da substância, assim como ao tempo de contato com a superfície corporal. Podem ocorrer complicações agudas, crônicas e até mesmo sequelas permanentes^(^ ^10^ ^)^. 

A queimadura química em crianças é primariamente acidental. Na revisão da literatura, só foram encontrados dois relatos de caso de exposição à soda cáustica como forma de maus-tratos à criança^(^ ^11^ ^,^ ^12^ ^)^. 

A ocorrência rara de lesões por contato causadas por esse agente químico, bem como o aspecto extenso e profundo da lesão no caso em questão, certamente contribuíram para que a equipe da emergência suspeitasse de abuso sexual. Seria pouco provável que apenas o contato com um produto de limpeza, conforme relatado pela mãe, fosse capaz de causar tal lesão. De fato, a incompatibilidade entre o tipo de lesão e o mecanismo relatado pelos responsáveis é um dos indicadores para a suspeita de violência e requer um aprofundamento da avaliação para esclarecer as circunstâncias que levaram à lesão^(^ ^13^ ^)^. 

Quando existe a possibilidade de a causa de uma lesão ser intencional, os profissionais se deparam com novas questões a serem esclarecidas, como no caso relatado: a suspeita de abuso sexual e a possibilidade de ter havido negligência na supervisão da criança. Ambas as situações colocam para a equipe de saúde o desafio de transcender às questões meramente clínicas para compreender os aspectos sociais, culturais e afetivos das famílias atendidas, o que requer disponibilidade interna dos profissionais para uma escuta diferenciada e certa experiência na condução dos casos de forma tranquila e sem assustar ou acusar a família. 

Esclarecer as circunstâncias que levaram à lesão, saber quem cuida da criança e se esses cuidados estão adequados para sua idade e seu desenvolvimento, como é o ambiente em que a família vive, se já ocorreram outros acidentes, se houve demora na procura de atendimento e se a criança interage e se comporta de forma adequada com o cuidador são alguns dos parâmetros que auxiliam nessa avaliação^(^ ^14^ ^)^. Dessa forma, pode-se verificar o grau de risco ao qual a criança pode estar exposta e decidir pela indicação de internação como medida protetiva, de notificação ao Conselho Tutelar, necessidade de seguimento ambulatorial, entre outras providências a serem tomadas. Cabe ressaltar que a notificação deve ser feita mesmo nos casos suspeitos, sempre que não haja possibilidade de acionar outros profissionais que auxiliem no esclarecimento dos indícios encontrados. 

Sendo a Emergência uma importante porta de entrada para a rede de saúde, cabe ainda aos profissionais que aí trabalham a tarefa de criar um vínculo positivo com os responsáveis, de modo que haja adesão ao seguimento proposto após a alta. O acompanhamento ambulatorial é uma forma de garantir a proteção da criança, na medida em que possibilita a supervisão do caso em longo prazo. Desta forma, pode-se conhecer o vínculo dos pais com a criança, o contexto familiar e a ocorrência de novos problemas de saúde, além de orientar os pais quanto à prevenção de acidentes e de maus-tratos. 

A negligência é um tipo de violência muito frequente contra crianças em todo o mundo. Segundo o Ministério da Saúde, caracteriza-se pelo descaso com o bem-estar e a segurança, com a afetividade ou com a educação de crianças ou adolescentes ou ainda pela recusa dos pais em aceitar as orientações do calendário vacinal, tratamentos medicamentosos, orientação educacional e preventiva^(^ ^13^ ^)^. A cronicidade dessas ações e sua reincidência são importantes características a serem avaliadas para configurar a presença de negligência. 

A história social dessa família, os vínculos familiares e os cuidados prévios com a criança em questão apontavam para cuidados adequados e indicavam que o acidente decorreu de um descuido pontual, passível nas circunstâncias apuradas. No seguimento ambulatorial, a mãe afirmou que costuma trancar a porta do quarto para dormir à tarde com a filha, mas havia se esquecido naquele dia. A equipe do ambulatório teve como foco a orientação para que a mãe acionasse sua rede de apoio familiar para ajudar nos cuidados da criança e reforçar as medidas de prevenção de acidentes. Nos seis meses seguintes ao acidente, houve boa adesão ao acompanhamento, sem qualquer tipo de intercorrência. 

Para promover a segurança de crianças de um a dois anos, é importante orientar os pais quanto a não deixar ao alcance os objetos pontiagudos, cortantes ou que possam ser engolidos; colocar proteção nas escadas e janelas; proteger os cantos dos móveis; colocar obstáculo na porta da cozinha e manter fechada a porta do banheiro; usar assento especial para crianças no automóvel, sendo este colocado no banco de trás do carro; guardar produtos de limpeza e remédios em armários trancados, colocando-os em lugares altos^(^ ^15^ ^)^. 

A ocorrência deste caso chama mais uma vez a atenção dos profissionais de saúde para a relevância da prevenção de acidentes, que deve ser feita rotineiramente durante todo o acompanhamento da criança e do adolescente, adaptando-se às etapas de desenvolvimento e ao contexto de vida das famílias. As equipes de saúde precisam também estar preparadas para incluir a possibilidade de violência como um diagnóstico diferencial quando pertinente, sabendo conduzir o esclarecimento das suspeitas de modo acolhedor, a fim de não prejudicar o próprio processo de cuidado e nem a relação com a família.
